# Effects of body-oriented yoga: a RCT study for patients with major depressive disorder

**DOI:** 10.1007/s00406-021-01277-5

**Published:** 2021-07-10

**Authors:** Miriam Bieber, Esra Görgülü, Daniela Schmidt, Kirsten Zabel, Semra Etyemez, Benedikt Friedrichs, David Prvulovic, Andreas Reif, Viola Oertel

**Affiliations:** 1 Department of Psychiatry, Psychosomatic Medicine and Psychotherapy, University Hospital of Frankfurt, Goethe University, Frankfurt am Main, Germany; 2 Department of Epidemiology, Johns Hopkins Bloomberg School of Public Health, Baltimore, USA; 3 Asklepios Hospital for Mental Health, Langen, Germany

**Keywords:** Depression, MDD, Yoga, Exercise, Intervention

## Abstract

The major depressive disorder is one of the most common mental illnesses worldwide. Current treatment standards recommend a combined therapy with medication and psychotherapy. As an additive component and to further improvements in treatment, physical activity such as yoga may be integrated into conventional treatment. This study investigates the impact of a 3-month body-oriented yoga in patients with major depressive disorder (MDD). In total, *n* = 83 patients were included. An intervention group received a vigorous Ashtanga-Yoga three times a week. The waiting-list control group obtained a treatment as usual (TAU). As a primary outcome depression scores (Beck Depression Inventory-II (BDI-II), Montgomery Asberg Depression Rating Scale (MADRS)) were tested at three time points. Secondary outcome was the positive and negative affect [Positive and Negative Affect Scale (PANAS)] and remission rates. To analyze the data, multilevel models and effect sizes were conducted. The results showed an improvement in BDI-II scores for both groups over time [*γ* =  −  3.46, *t*(165) =  − 7.99, *p* < 0.001] but not between groups [*γ* = 0.98, *t*(164) = 1.12, *p* = 0.263]. An interaction effect (time x group) occurred for MADRS [*γ* = 2.10, *t*(164) = 2.10, *p* < 0.038]. Positive affects improved over time for both groups [*γ* = 1.65, *t*(165) = 4.03, *p* < 0.001]. Negative affects decreased for all over time [*γ* =  −  1.00, *t*(165) = − 2.51, *p* = 0.013]. There were no significant group differences in PANAS. Post hoc tests revealed a greater symptom reduction within the first 6 weeks for all measurements. The effect sizes for depression scores showed a positive trend. Remission rates indicated a significant improvement in the yoga group (BDI-II: 46.81%, MADRS: 17.02%) compared to the control group (BDI: 33.33%, MADRS: 8.33%). The findings suggest that there is a trendsetting additive effect of Ashtanga-Yoga after 3 months on psychopathology and mood with a greater improvement at the beginning of the intervention. Further research in this field can help to achieve more differentiated results.

**Supplementary Information:**

The online version contains supplementary material available at 10.1007/s00406-021-01277-5.

## Introduction

Affective disorders have a high prevalence worldwide in the general population [1] with more than 40.27 million people in the European region suffering from depressive syndromes [1, 2]. The treatment of depressive disorders is broadly researched [3, 4]. The gold-standard method provides pharmacological therapy and psychotherapy for a major depressive disorder (MDD) [5]. However, despite well-researched treatment methods, many patients experience insufficient or non-response [6]. In addition, a lot of individuals show considerable residual symptoms despite treatment according to the guidelines [7]. Side effects caused by medication can severely restrict adherence [8]. Access to psychotherapy is also difficult for many patients due to long waiting lists, own prejudices or fears and stigmatization [9]. Also, the prevention of recurrence and the treatment of chronic courses are limited by previous treatment methods in several patients [10]. For these people, it is important to establish alternative concepts in addition to conventional treatment methods.

In this context, research addresses the integration of physical activity and mindfulness-based treatments in the treatment of MDD. Recently, a range of exercise-therapy as well as for depression have been included in the guidelines [11]. Many interventional trials have so far demonstrated that regular physical activity has a reducing effect on depressive symptoms [4, 12–15] in addition to positive physical benefits, and that exercise improves the general well-being in the case of psychological disorders [16, 17]. In addition to conventional cardio training, yoga as a form of exercise is in the focus of scientific research [18, 19]. Yoga seems to be an alternative form of physical exercise in the treatment of psychological disorders and offers advantages in terms of adherence due to its increasing popularity in the western world.

The yoga philosophy pursues a connection of body and mind [20, 21]. The aim is to bring harmony between the mental and physical state, to relax the body and to reduce maladaptive mental activity like rumination [21]. Yoga combines mindfulness-based elements with breathing techniques and specific movements and stretching [21, 22]. Depending on the type of yoga being practiced, the mindfulness-based part or the physically demanding part is in the foreground [22]. Studies on healthy individuals show the wide-ranging effects of yoga-like reduced body strains, lower levels of anxiety and self-reported stress [23]. In fact, regular yoga practice improves general well-being and has a balancing effect on mood [24, 25]. With regard to people with mental or somatic illness, studies with cancer patients [26], patients with chronic pain [27] or depression [28, 29], positive health effects have been shown through regular yoga practice.

The number of studies concerning yoga in the treatment of depressive disorders increased, and reviews and meta-analyses attempt to combine the partly heterogeneous study designs and results to provide reliable information [30]. Up to date, several studies revealed positive results about the effectiveness of yoga in the treatment of depression. In a randomized controlled trial, a 6-week yoga intervention in depressed and anxious participants was compared to a waiting-list control group [31]. There were statistically significant differences between the yoga and the control group concerning the reduction of depression values which leads to the conclusion that a combination of yoga and usual treatment methods is more effective for depression than the usual treatment on its own. An 8-week intervention study with yoga practice in depressed individuals showed positive effects on physical well-being and mood with increasing positive affect and reduced negative affect [24]. Recent treatment trials with yoga interventions achieve similar results and show an at least additive effect of yoga in the treatment of depression [16, 32, 33]. Another RCT study evaluated mindfulness-based yoga in patients over 12 weeks [34]. Here, depressed women received a yoga home treatment twice a week and a control group were instructed to do a walking-based workout. The findings showed that over time depressive symptoms were reduced for both groups. A difference between the groups could not be determined. These results are in line with other studies that have not successfully proved the superiority of yoga or an add-on effect [35]. Therefore, the literature seems to be inconsistent in positive effects of yoga. There is a need for more trials in this area due to some methodological limitations in existing studies.

However, many studies do not define in their intervention measures the yoga style or the teaching methods [36, 37]. It should be noted that many studies include yoga in the field of mindfulness [38, 39] and the focus is especially on symptoms such as rumination or low self-esteem, which can be improved through mindfulness, non-judgmental practice or acceptance training. However, the physical component, which is unquestionably also influenced by yoga, is given little consideration, and is not addressed by the low physical intensity of mindfulness-based yoga training. There is a lack of studies investigating whether more vigorous forms of yoga can have a higher impact on depressive symptoms and whether physiologically mediated effects can reduce depression symptoms.

The Ashtanga-Yoga used in the present study includes mindfulness-based elements, breathing techniques and especially physically demanding exercises, which provide enhanced physical activity compared to other forms of yoga. Since studies on aerobic training have shown positive effects in the therapy of depression [40–42], these physiological processes could also be induced in a physically demanding yoga style at an aerobic level. It is, therefore, necessary to investigate whether the positive effects identified to date cannot be attributed solely to the mindfulness-based approach.

Preliminary findings show that yoga adjusts physiological imbalances in the depressed brain and body. Regular yoga practice shows a stabilization of the immune defense and an improvement in sleep [43, 44]. Yoga can impact the endocrine system by lowering the activity of the hypothalamic–pituitary–adrenal axis and thereby reducing the production of the stress hormone cortisol [45, 46]. Positive outcomes of yoga can also be noticed on cognitive performance because of an increase in neurogenesis involved neurotransmitters, the brain-derived neurotrophic factor (BDNF) [44, 47]. This has a neuroplastic effect and ensures the formation of new neurons [44]. Moderate-to-high effects on attention, speed of processing, executive functions and memory could be found [48, 49]. However, it remains unclear from the reported results to which extent increased physical activation causes antidepressive effects. The additional benefit of this study is mainly based on a yoga practice which is highly standardized and comparable to aerobic training. Ashtanga-Yoga is based on a physically demanding, stringently practicing sequences of exercises, which are always repeated in the same order [50, 51]. In a scientific context, this ensures high standardization, reproducibility, and comparability of the intervention. In addition to a long intervention period of 12 weeks with three measurement time points and a sample of patients diagnosed with MDD, this study offers further evidence of the effectiveness of a body-oriented, vigorous yoga.

The aim was to determine whether regular Ashtanga-Yoga reduces depression scores, supports remission, and regulates mood at three different time points. Thereby the focus was on possible effects that can be achieved in addition to a treatment as usual (TAU) for a yoga-practicing group compared to a control group. As a primary outcome we expected a reduction in BDI-II and MADRS scores. Secondary outcome were remission rates for both groups and test scores in the PANAS positive and negative. We expected improved values for both groups caused by the TAU but with superiority of the yoga group in each parameter.

## Materials and methods

### Overview, study design and sample

The data presented here are part of a mono-center, prospective, controlled parallel-trial-study on exercise and physical activity in psychiatric patients at the Department of Psychiatry, Goethe-University Frankfurt, Germany. The study was reviewed and approached of the ethics committee (registration-number 60/15, 18.05.2015), safety risks were controlled. Primary outcome was the change in the depression scores Beck Depression Inventory-II (BDI-II) and Montgomery Asberg Depression Rating Scale (MADRS). Secondary outcome was the Positive and Negative Affect Scale (PANAS) as well as remission rates.

Allocation and Randomisation process included outpatients diagnosed with MDD. They got detailed study information and the written informed consent. All diagnostic inclusion criteria such as the diagnosis of MDD as well as psychological and somatic exclusion criteria were checked (detailed criteria in section participants).

If all necessary inclusion criteria were fulfilled and no exclusion criteria were present, the patients were randomised. A double-blind randomisation was carried out using number generation by Excel in a ratio of 1:1 into the intervention or control group. After allocation, the patients were informed about the group to which they were assigned. The intervention started 3 weeks after randomisation. In the meantime, further psychological and physiological examinations were carried out. There were drop outs in both groups due to necessary medication changes, physiological complaints or premature termination of study participation by patients.

**Fig. 1:**
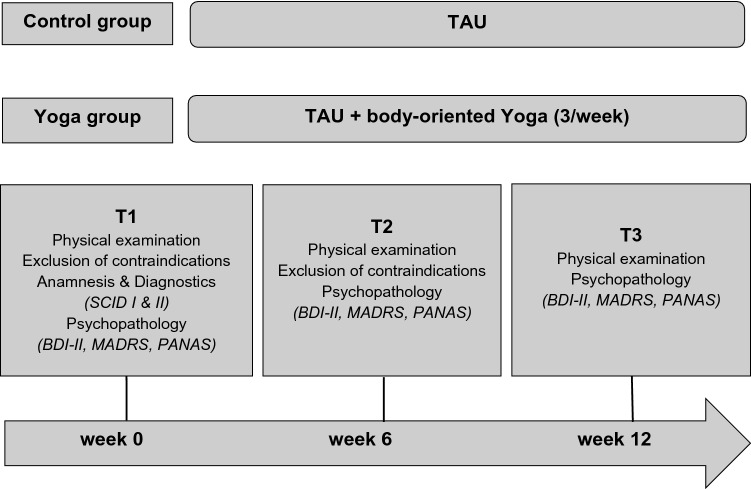
Illustration of the study design and determinants of treatment

Intervention group (yoga group) participated in a guided yoga class added to a TAU of current psychopharmacology and psychotherapy (see Fig. 1). The other group was a waiting-list control group (control group) with TAU and no additional exercises. The intervention period lasted three months. A baseline survey at Time point 1 (T1) with a detailed psychiatric anamnesis, questionnaires and physical examination was carried out before the start of the intervention to determine the current psychopathology with symptom severity and to exclude psychological and physical contraindications. At time point 2 after 6 weeks (T2), further psychological surveys were carried out with a renewed examination of the physical and psychological prerequisites for participation. At time point 3 after 12 weeks (T3), the intervention phase was completed with a final survey. During the entire study period, all patients were monitored by the medical care team to be able to address possible exacerbations of the physical or mental condition or to avoid or react to adverse effects. All data were collected and analysed by blinded assessors. The outcome focused on severity of depression symptoms, remission, and changes in positive and negative affect.

**Fig. 2:**
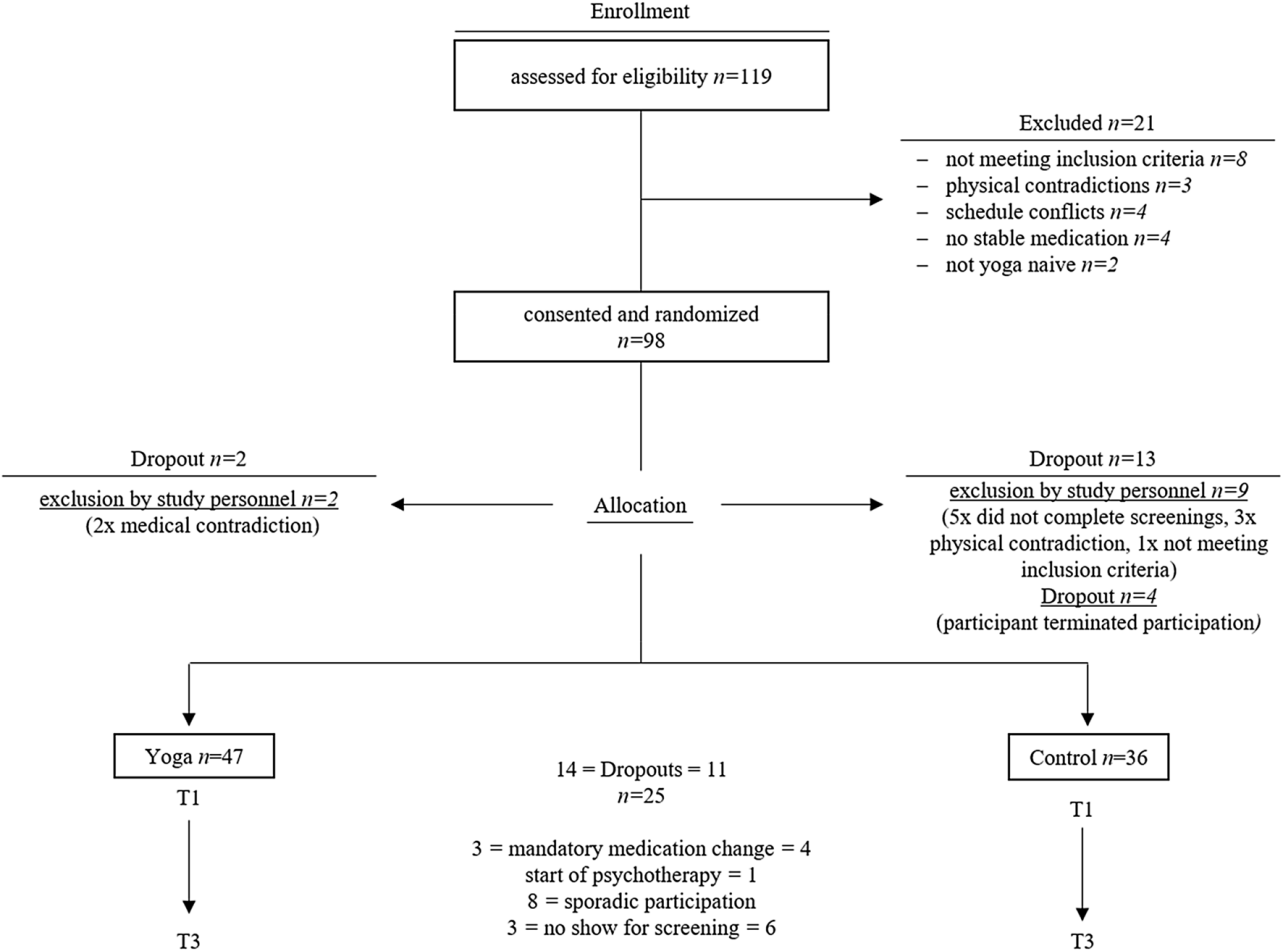
Sampling and participant flowchart (data collection from 2015 to 2016)

Figure 2 shows an overview of the sample size and the recruitment process. We identified 119 potentially eligible participants of whom 83 were able to participate after checking the inclusion criteria, contraindications and due to some dropouts caused by organizational problems and individual circumstances. The dropout rate showed no significant difference between the groups. We use the phrase “dropout” in this context as an overall term for two different types of participant exclusions from the study. On one hand, the term dropout refers to the exclusion of subjects by the study personnel due to a lack of inclusion criteria or the fulfilment of exclusion criteria. On the other hand, dropout refers to patients who have independently decided to terminate their participation in the study (patients excluded by study personnel).

Over the measurement periods, 25 subjects (30.12%) had to be excluded due to mandatory medication changes, the start of psychotherapy, sporadic participation and missing out at the screenings. An intention to treat approach (ITT) was chosen for the preparation of the data, which maintains the number of cases at the baseline level through substitution of mean values.

### Participants

The study included 66 females and 17 males (see Table 1) with MDD. All participants were outpatients and were tested with the SCID-I and II of the Diagnostic and Statistical Manual of Mental Disorders IV (DSM-IV, German version) [52] and screened with the BDI-II [53] (BDI-II at baseline for yoga-group: 23.19 (min. 20.00, max. 41.00), for control-group: 26.08 (min. 20.00, max. 51.00), BDI Cut Off ≥ 14) to ensure the diagnosis of MDD. Further eligibility criteria included: age 18–65 years; no experiences in yoga or any other mindfulness or body-oriented treatment; fluent in German language and able to manage the questionnaires; able to participate in the yoga classes three times a week for 12 weeks; stable medication for at least 2 months before the start of the study. All participants were physically examined whether they could participate at the yoga sessions in the beginning and monitored during the intervention to check the current health status. The exclusion criteria were as follows: patients under 18 years and over 65 years, patients with comorbid psychiatric disorders as well as acute psychosis, personality disorders, illegal drug abuse and substance use disorders; physical contradictions which did not allow participating in the intervention, such as cardio-vascular diseases, respiratory symptoms and orthopaedical restrictions; pharmacological modifications during the intervention period, the initiation of psychotherapy or any body-oriented or mindfulness treatments up to 2 months before and during the intervention.

**Table 1:** Sociodemographic and clinical characteristics of study participants by subgroup

	Total (*N* = 83)	Yoga group (*N* = 47)	Control group (*N* = 36)
Sociodemographic characteristics			
Age	*M* = 49.65; SD = 9.83	*M* = 48.38; SD = 10.21	*M* = 51.31; SD = 9.19
Gender (female: male)	66: 17	38: 9	28: 8
Nationality:			
German	90.36%	87.23%	94.44%
Other	9.64%	12.77%	5.56%
Years of education	*M* = 14.74; SD = 3.06	*M* = 15.23; SD = 3.27	*M* = 13.72; SD = 2.44
Psychiatric and physiological characteristics			
Duration of disease	*M* = 9.02; SD = 7.40	*M* = 8.79; SD = 8.19	*M* = 9.31; SD = 6.30
Number of previous depressive episodes	*M* = 2.69; SD = 1.25	*M* = 2.85; SD = 12.00	*M* = 2.50; SD = 1.16
Medication	75.90%	76.60%	75.00%
Psychotherapy	92.8%	91.49%	94.4%
BMI	*M* = 26.80; SD = 5.29	*M* = 26.98; SD = 5.47	*M* = 26.56; SD = 6.00

*M* mean difference, *SD* standard deviation, *p* significance, *p* ≤ 0.005, *BMI* body mass index

### Testing and instruments

At the beginning individuals received information on safety, instructions to the research and assigned the written informed consent for participation. Afterwards, the patients completed a psychological battery of tests which was carried out by a psychologist who was not involved in the study. All instruments were used in the German language.

BDI-II: The Beck Depression Inventory-II [53] is a screening instrument for self-assessment of depressive syndromes with 21 questions that can be answered on a graded scale with four possible answers. The sum value provides information about the possible presence of depressive symptoms.

MADRS: Montgomery Asberg Depression Rating Scale [54] is an external assessment questionnaire for evaluating the severity of a depressive syndrome. The semi-structured interview was conducted by blinded psychologists within the department. It consists of ten questions which refer to the period of the previous week and are evaluated on a scale with seven possible answers. The total score can be between 0 and 60, with a clinically relevant cut off ≥ 7.

PANAS: The Positive and Negative Affect Scale [55] is a self-report questionnaire for the assessment of individual positive and negative affectivity. It consists of 2 scales with 20 emotion-items. Each item can be assessed on a scale from 1 (not at all) to 5 (very much). The result is the sum of the values.

### Intervention

#### Yoga-exercise

Yoga classes had a duration of 90 min each and were held in groups of 10–12 patients. Sessions were executed three times per week and held for 12 weeks. If regular participation in 2 consecutive weeks was not guaranteed, the participant was excluded from the study set-up. The yoga exercises consist of sequences of body-oriented Ashtanga-Yoga [51], which represented fixed sequences and were repeated every session. All yoga classes were guided by a licensed yoga instructor who performed all asanas and supported participants during their lessons with verbal announcements and hands-on assists. The yoga sessions are divided into a warm-up period (15 min) consisting of sun salutations and stretching exercises, in the following, a cardio period (60 min) where participants performed dynamic and active yoga practice with more physical strain. As a cool-down (15 min) sun salutations and stretching were implemented as well as breath-controlling and meditation-elements. The training ensured that every participant was able to follow and to move in the prescribed way. For this purpose, exercises at different levels of complexity were offered, which could be adapted to the performance level of the participant.

#### Waiting-list control group

A waiting-list control group was implemented to identify possible effects clearly caused by the yoga intervention. Patients had the option to participate in a yoga session after waiting time. The control group received a TAU, consisting of antidepressant medication and psychotherapy.

### Statistical analysis

**Fig. 3:**
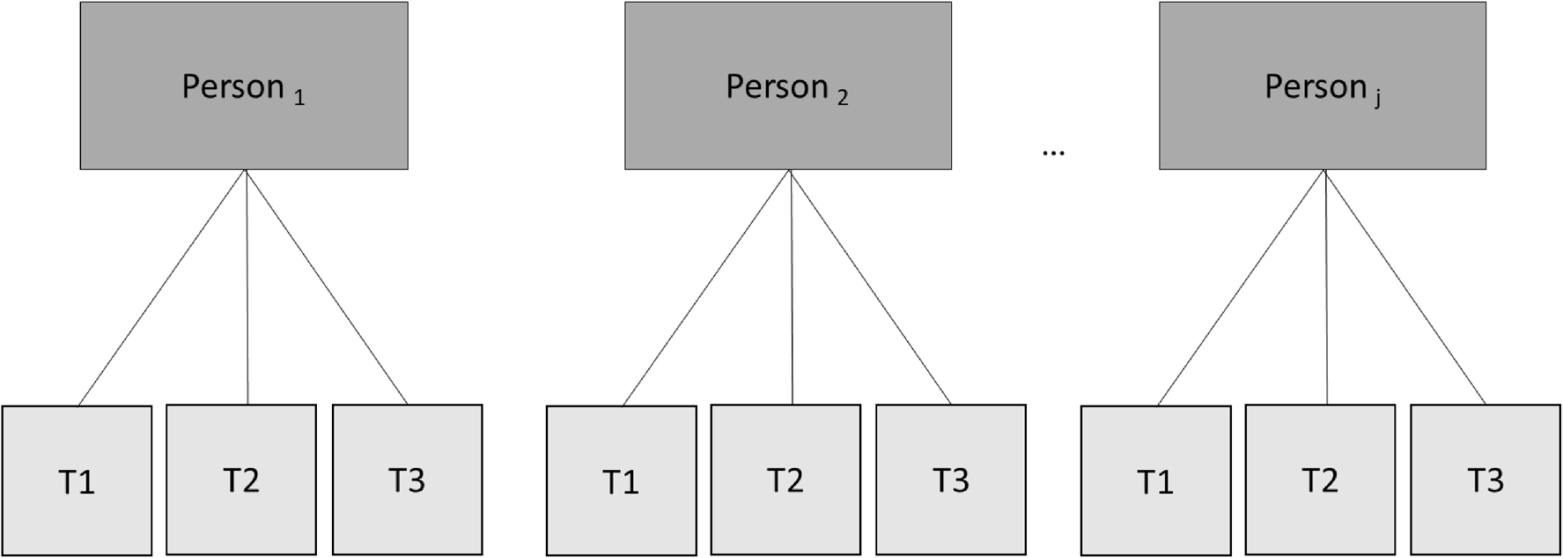
Hierarchical data structure of the multi-level model

For statistical analysis, data were conducted in a blinded manner using the programs SPSS 23 and R3.6.2. For all applied analyses for significance a two-tailed test with a *p* < 0.05 value was assumed. An intention-to-treat (ITT) approach was chosen to prepare the data structure. After descriptive evaluation, the data collected for the parameters of interest were analyzed at three measurement time points. The data structure presented here is nested, respectively, and consists of several levels. For the evaluation of nested data, so-called linear mixed models are used, which are less sensitive to outliers and violations of variance homogeneity [56]. Multi-level models which represent likelihood ratio tests, are more robust estimation models with an improved error analysis. They are mainly used for the evaluation of data with unequal group sizes. A nesting of data already arises during the data collection, which was collected in several steps. This type of data collection results in a so-called interdependence of the measured values. This dependency means that the measured values of persons in a group are more like each other than the data of persons between two different groups. Classical regression methods, however, assume that measurement errors are independent of each other [57]. If this assumption is not fulfilled, the results from the classical methods can no longer be reliably interpreted. A case that generates nested data is the repeated measurement. In this case, the measurement times are nested in persons [57]. This means that in the repeated measurement two values of a person are more like each other than the measured data of two different subjects. Repeated measurements are, therefore, generally dependent on each other. Traditionally, a repeated analysis of variance has been used when several measurement times are available. However, this has several disadvantages compared to multi-level models: it is sensitive to violations of variance homogeneity, the group sizes (or measurement times) should be different and it is sensitive to outlier values [56]. Therefore, multi-level analysis is also the method of first choice in this case, if longitudinal average data has to be investigated [56]. In the case of a measurement repetition, the repeated measurement values are at level 1 (L1) and the person is at level 2 (L2) [57] (see Fig. 3). The analysis initially provides for the creation of a zero model. To examine the hierarchical structure of the data, the ICC was computed. In this model, the variance components for the group-specific deviation from the mean value (intercept) and for the person-specific deviation are determined. These components can be used to determine the intraclass correlation (ICC) [57]. The ICC allows a prediction to be made about the size of the variance component at L1, which is explained by L2. If this value is substantially higher than 0, a nesting of the data can be assumed, and a multi-level model becomes necessary. This is always given in the case of a repeated measurement (Fig. 3).

**Fig. 4:**
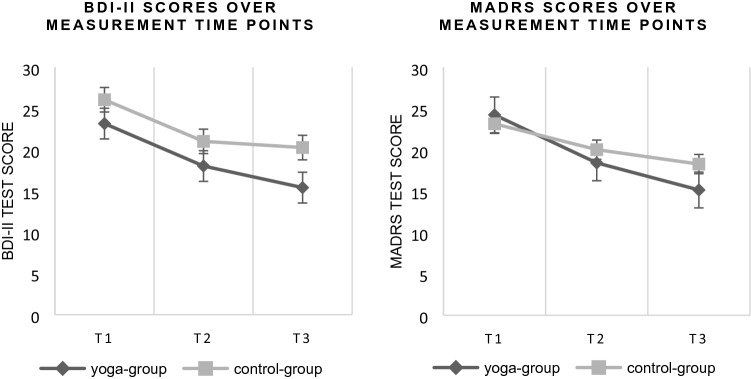
Charts of BDI-II and MADRS mean scores over measurement time points T1 to T3 for the yoga group (black line) and the control group (grey line)

In the next analysis steps, basic models such as the Random Intercept Model (RI) and the Random Slope Model (RS) get analyzed. The central distinction is that the RI model does not model deviations for the slope. In the RS model, on the other hand, it is assumed that there are also deviations from the mean slope. In the following, the RS and the RI models are compared with each other and the most restrictive model is selected. After model testing, a model inspection is performed. The inspection of assumption comprised several steps: testing for variance homogeneity and testing the normal distribution of residuals and linearity. (The Model equation is added to supplemental material. *R*^2^ calculated according to Nakagawa, Johnson, and Schielzeth, reporting the marginal *R*^2^ [58].)

In addition, Cohen’s *d* was calculated using differences in the mean values over all time points. Finally, a sensitivity analysis was carried out for detected effects in the yoga group, the control group and for the group comparison. The sensitivity analysis aimed to investigate whether the effects are robust to the error probabilities of alpha and beta due to the selected procedure and the sample [59]. As the individual comparisons are multiple tests, the Bonferroni correction [60] was used. Here, the alpha-error probability is divided by the number of tests. In the current case, three individual comparisons are performed (three measurement time points), therefore, 0.05/3 is calculated. The sensitivity is, therefore, determined for an alpha-level of 0.017.

## Results

### Mean change scores

Descriptive results showed a significant improvement in the yoga group in the severity of depression, but the control group also improved their symptoms (see Table 2). For BDI-II scores, the mean change score over the intervention period of 12 weeks declined by 7.77 points [*t*(162) = 4.52, SE = 1.14, *p* < 0.001] for the yoga group compared to the control group with a decline by 5.81 points [*t*(162) = 4.46, SE = 1.30, *p* < 0.001]. The MADRS scores showed a reduction of 9.09 points [*t*(162) = 6.83, SE = 1.33, *p* < 0.001] in the yoga group and 4.89 points [*t*(162) = 3.21, SE = 1.52, *p* < 0.005] in the control group. For the PANAS positive, a negative indicator showed that the extent of positive affects in both groups changed by -3.11 points [*t*(162) = 4–2.85, SE = 1.09, *p* = 0.014] for the yoga group and by − 3.53 [*t*(162) = − 2.83, SE = 1.25, *p* = 0.014] in the control group over 12 weeks. The PANAS negative showed a greater reduction of 2.55 points [*t*(162) = 2.41, SE = 1.06, *p* = 0.045] for the yoga group compared to the control group with 1.29 points [*t*(162) = 1.06, SE = 1.21, *p* = 0.539]. Figure 4 shows charts of BDI-II and MADRS mean scores over measurement time points.

**Table 2:** Mean scores with standard deviation and mean change scores of outcome measurements of BDI-II, MADRS and PANAS

	Yoga group				Control group			
Outcome measure	Time point 1 M (SD)	Time point 2 M (SD)	Time point 3 M (SD)	Mean change score	Time point 1 M (SD)	Time point 2 M (SD)	Time point 3 M (SD)	Mean change score
BDI-II	23.19 (11.36)	18.04 (11.66)	15.42* (11.29)	7.77***	26.08 (13.07)	21.30 (11.38)	20.28 (12.19)	5.81***
MADRS	24.26 (8.09)	18.45 (10.28)	15.25* (7.39)	9.09***	23.18 (10.29)	20.05 (9.03)	18.30 (7.61)	4.89*
PANAS								
positive	24.80 (9.11)	27.42 (8.23)	27.90* (8.74)	− 3.11*	22.17 (7.28)	24.35 (7.63)	25.70 (7.72)	− 3.53*
PANAS								
negative	23.16 (8.23)	20.44 (6.75)	20.61* (7.73)	2.55*	23.94 (9.41)	22.52 (7.97)	22.66 (7.71)	1.29

*M* mean difference, *SD* standard deviation

^a^Significant difference *p* ≤ 0.005

^b^Significant difference *p* ≤ 0.001

### Multi-level models

#### Primary outcome: BDI-II

For the BDI-II, the ICC was 0.61. The model comparison showed that the random intercept model fits the data equally well, [*χ*^2^(2) = 2.73, *p* = 0.256], so RI model was applied. The *R*^2^ for this model was 0.07. There is a significant mean difference after 3 months (*γ* =  − 3.46, SE = 0.43, *t*(165) = − 7.99, *p* < 0.001) for all participants. The adjusted *p* value (Bonferroni) [61] remained significant (*p* < 0.001).

No significant difference was found between the groups over time (*γ* = 0.98, SE = 0.87, *t*(164) = 1.12, *p* = 0.263). In the post hoc individual comparison, a 100% negative slope was found, which is equivalent to a decrease in depressive symptoms in all subjects. A closer look revealed a significant reduction of the BDI-II values from T1 to T2 for the yoga group (Δ12 = 5.15, SE = 1.14, *t*(162) = 4.52*, p* < 0.001) as well as the control group (Δ12 = 4.78, SE = 1.30, *t*(162) = 3.67, *p* < 0.001). A later change between T2 and T3 was significant but with a lower intensity than within the first 6 weeks for all subjects (*γ* = 3.07, SE = 1.48, *t*(164) = 2.07, *p* = 0.040].

#### Primary outcome: MADRS

The RI model was chosen for the data of the MADRS^1^ with an ICC = 0.43. The *R*^2^ for this model was 0.104. Here, after 3 months, the depression parameters for the yoga group were descriptively lower compared to the control group. On average, a significant change over time can be observed for both groups [*γ* =  −  3.63, SE = 0.51, *t*(165) = − 7.19, *p* < 0.001], even the Bonferroni adjusted *p* value was significant (*p* < 0.001).

When considering the reduction of values within the time period of the study, a significant change [*γ* =  −  4.54, SE = 0.66, *t*(164) = − 6.84, *p* < 0.001] can be observed for the yoga group, whereas the control group showed a less significant reduction of the depression parameter [*γ* = 2.10, *SE* = 1.01, *t*(164) = 2.09, *p* = 0.039]. On the other hand, a significant interaction effect was observed with consideration of the group and the time [*γ* = 2.10, SE = 1.01, *t*(164) = 2.10, *p* < 0.038] with a stronger symptom reduction in the yoga group. Results of the individual change showed a negative sign for the slope, which suggests that each subject reduced the MADRS value over time. Upon closer examination of the measurement points between T1 and T2 a significant reduction in depression symptoms could only be shown for the yoga group [Δ = 5.81, SE = 1.33, *t*(162) = 4.36, *p* < 0.001, control group: Δ = 3.13, SE = 1.52, *t*(162) = 2.07, *p* < 0.102].

The results showed that no significant change beyond the previous change between T2 and T3 was obtained [*γ* = 2.02, SE = 1.75, *t*(164) = 1.16, *p* = 0.249].

#### Secondary outcome: PANAS

Results of the PANAS positive showed an ICC = 0.53. Considering the chosen RI model^2^ (*R*^2^ = 0.049), there was no significant change between the groups [*γ* = 0.21, SE = 0.83, *t*(164) = 0.26, *p* = 0.798]. A significant difference could be observed over the measurement time T1 to T3 [*γ* = 1.65, SE = 0.41, *t*(165) = 4.03, *p* < 0.001]. [Bonferroni adjusted *p* values (*p* = 0.000010)]. Individual change showed a positive sign of 97.59%, which corresponds to an improvement in the value. Post hoc, the yoga group showed a significant improvement in PANAS positive between T1 and T2 [Δ = − 2.63, SE = 1.09, *t*(162) = − 2.41, *p* = 0.045], whereas the control group did not [Δ = − 2.18, SE = 1.25, *t*(162) = − 1.75, *p* = 0.189]. It turned out that the change between T2 and T3 was not significant at all, [*γ* =  − 1.58, SE = 1.41, *t*(164) =  −  1.12, *p* = 0.265].

PANAS negative data showed an ICC = 0.54, so a RI model was applied [*χ*^2^(2) = 5.499, *p* = 0.06396]. The *R*^2^ for this model was 0.02. The change over time was significant for both groups [γ =  −  1.00, SE = 0.40, *t*(165) = − 2.51, *p* = 0.013]. Adjusted *p* value was significant [*p* = 0.05].

There is no significant difference between the groups [*γ* = 0.63, SE = 0.81, *t*(164) = 0.79, *p* = 0.433]. The individual change results in a negative sign for 73.49%, which may be attributed to a reduction of the PANAS negative. The negative affect decreased significantly towards T2 in the yoga group [Δ = 2.72, SE = 1.06, *t*(162) = − 2.57, *p* = 0.003], but not in the control group [Δ = 1.42, SE = 1.21, *t*(162) = 1.75, *p* = 0.47]. Between T2 and T3, there was no significant change [*γ* = 2.31, SE = 1.38, *t*(164) = 1.68, *p* = 0.095].

#### Secondary outcome: remission rates

Remission rates for the ITT-population were conducted with a cut off for the BDI-II ≤ 12 [62], at the end of the treatment. The yoga group showed a remission rate of 46.81% with 22 patients who no longer report any clinically relevant symptoms. The control group had a remission rate of 33.33% with 12 patients who have remitted. This difference was shown as significant [*t* = 29.285, df = 82, *p* < 0.001]. (Bonferroni adjusted *p* value *p* < 0.001).

For the data of MADRS, a cut off MADRS ≤ 7 [62] was used to consider the remission rate. For the yoga group, a remission rate of 17.02% with eight patients without symptoms and 8.33% for the control group with three participants were found. Results were statistically significant [*t* = 49.874, df = 82, *p* < 0.001], even adjusted *p* values (*p* < 0.001).

### Effect size Cohen’s *d*

Cohen’s *d* analyses were used to calculate the effect sizes [63] of the difference in outcome variables between different time points per group. In the case of repeated measurement data, an average effect size from *d* = 0.35 and a high effect size from *d* = 0.57 was mentioned. Table 3 shows the effect sizes at the different time points of the two groups. The analysis showed higher effect sizes for the yoga group for the depression-specific instruments BDI-II and MADRS than in the control group. Therefore, the BDI-II for the yoga group showed a high effect size between T1 and T2 and over the entire intervention period of 12 weeks. Between T2 and T3, the effect sizes in both groups were low. High effect sizes for the MADRS can be determined in the yoga group between T1 and T2 and between T1 and T3. The control group, in contrast, showed a high effect only around the entire 12 weeks from T1 to T3, whereby this remains below the values of the yoga group. For the PANAS positive, average effects are obtained for the control group between T1 and T3 and between T2 and T3. The PANAS negative also showed an average effect size only in the control group over the entire measurement period.

**Table 3:** Effect sizes Cohen’s *d* for differences between time points

		Cohen’s *d*			
Group	Time point	BDI-II	MADRS	PANAS-pos	PANAS-neg
Yoga group	1–2	0.768	0.521	0.324	0.382
	1–3	0.885	0.898	0.347	0.320
	2–3	0.290	0.382	0.063	0.021
Control group	1–2	0.669	0.398	0.356	0.263
	1–3	0.717	0.650	0.569	0.168
	2–3	0.161	0.219	0.203	0.020

Using differences of mean scores to calculate effect sizes

Regarding the sensitivity analysis for the sample of the yoga group, effects with a size of *d* = 0.44 can be detected with a probability of 80%. For the control group, only larger effects from *d* = 0.51 could be detected with 80% probability. If the difference is smaller, the power decreases proportionally. For the comparison of the groups, it can be determined that there was sufficient power to detect effects from *d* = 0.66.

## Discussion

The present study examined whether regular yoga practice can reduce depressive symptoms and lead to improvements in mood. Due to the existing conventional treatment with medication and psychotherapy, a reduction of depressive symptoms was expected for both groups, however, for the group with regular yoga to a more substantial extent. The results showed that patients with regular yoga training experienced a slightly greater improvement in depressive symptoms and mood, and this trend indicates that yoga as an additive method should be more widely used in treatment. Any adverse events were registered for the entire study interval and discussed with the study physicians. No non-interventional adverse of the intervention were found. There were no safety risks for the participants during the intervention.

### Depressive symptoms

Both groups reduced their BDI-II scores over time. MADRS scores showed an interaction effect for the group and the time point with a greater symptom reduction in the yoga group. Remission rates support this trend with a significant difference between the groups. At the end of the intervention, almost half of the patients in the yoga group showed no clinically relevant symptoms, whereas in the control group, only a third exhibited relevant depressive symptoms. Furthermore, the effect sizes for the yoga group showed a stronger improvement in depression severity compared to the control group, which supports previous research [16]. With a closer look at the measurement time points, there is a greater change from T1 to T2 for the yoga group, which indicates a faster response, whereas for the MADRS, the control group did not show a significant improvement in symptoms in the first 6 weeks. Also, between the second and third measurement time point, the yoga group showed a greater decrease in symptoms. The effect sizes suggested high effects for the yoga group, while the values of the control group remained lower. In contradiction to previous findings, which assume an antidepressant effect of yoga to occur later in the intervention period [30], it is noticeable that in our study, the greater change in the yoga group was in the first 6 weeks. This study was able to show that yoga does not work with a prolonged time lag as often described in the literature. Within the first 6 weeks, there is a significant reduction of symptoms, which indicates a fast response to the intervention. This suggests that yoga may provide rapid symptom improvement, especially at the beginning of a depression treatment and before a TAU can have any effect. It is possible that the vigorous Ashtanga-Yoga can cause this effect due to the higher aerobic level, compared to the mindfulness-based yoga treatments with later effects [30]. An explanation of our early effect could also be a psychological one like a high level of motivation at the beginning and the intention to increase physical effort and expenditure of time. It can also be assumed that a stronger physical adaptation process takes place, especially at the onset of training, which has positive consequences in terms of a reduction in symptoms. This effect may be weakened during the intervention, or regular participation and willingness to exert decrease due to decreasing motivation.

### Positive and negative affect

The effect of yoga on the positive and negative affect measured in the PANAS has already been shown in a previous study [26], which was not replicated in this study. Although descriptively improved values were found for PANAS positive in the yoga group, no significant difference to the control group could be determined. The PANAS negative even showed an increase in negative affect in 25.51% of all participants. It can be assumed that regular yoga slightly increased the rate of positive affect, but the rate of negative affect is only partially influenced, and a negative affective state is still predominant in many cases. It should be noted that in treatment trials with more positive results concerning mood changes [24] healthy subjects without psychological limitations were included and the study design was conducted differently with considerably more measurement points. This may lead to the conclusion, that more frequent measurements are necessary for a valid representation of mood changes. However, the results suggest that in both groups the values for the negative affect have slightly decreased and those for the positive affect have increased, which can indicate a tendency towards change in affect in both groups.

### Limitations

The present study shows some statistical and methodological limitations which contradicts a clear conclusion about the effectiveness of yoga on depressive symptoms. A lack of a fully randomized study design can distort the results. Due to organizational problems at the start of the enrollment and the study set-up, randomization of the groups is viewed critically. Groups have no equal sizes. Dropouts and exclusion from the trial by study personnel in the control group before the start of the study were caused by premature discontinuation due to lack of compliance and disappointment with group allocation as well as medical contradictions.

Several limitations were also revealed in the sensitivity analysis. For the yoga group, the sensitivity is therefore sufficient to detect moderate (medium: 0.35, large: 0.57) effects over time. Accordingly, there is a lack of power to secure the detection of the identified effects. Since the groups are not of equal size, the power to detect effects within the two groups is not similar. For the control group, this indicates that there is a lack of power and that smaller changes (i.e., changes comparable to those in the yoga group) cannot be found. For the group comparison, there is an obvious lack of power, even if minor differences were already relevant.

Further conflicts arise from the control group receiving a TAU. Possible ceiling effects and an overall lower treatment effect can be expected. It has been shown that patients who are already undergoing treatment and are referred to yoga intervention by the health care system have less benefit from the intervention than patients without previous treatment [29]. Because of the previous criticism of the studies cited and with regard to the results in the present study, it can be assumed that the effects in the non-therapeutically treated participants are more likely to be related to less severe or subclinical depression than to the treatment context.

Another point that has to be mentioned is the sample size, which was further reduced by dropouts. Adherence in intervention studies with movement components is a general problem and is also reflected in other publications [30, 64, 65]. In addition, adherence decreases with increasing depressiveness [65]. High dropout levels can lead to reduced statistical performance, which can underestimate results [66]. Especially, the long intervention time in our study requires constant motivation and a certain level of vigor and abilities in everyday life, which is often limited in the context of depression. The 12-weeks intervention represents a rather long duration compared to other studies [18, 30]. Only a few studies offered a yoga intervention that has a duration of similarly 12 weeks [67, 68]. It is now necessary to examine which duration and frequency are appropriate. According to our results, symptom reductions already showed up quickly within the first weeks, the further effects prove to be moderate. For physical training in the endurance range there are already general recommendations regarding duration and frequency. This is not well established in the new field of yoga and needs to be researched further [67]. Based on our results and experiences in this study, we recommend a physically demanding yoga at aerobic level to receive a fast response. We recommend training at least 1 to 3 days per week to build up a routine especially in the beginning and to ensure progress. The group setting can have a motivating and supporting effect, while instructions by a yoga teacher prevent incorrect postures and can be a further motivational factor.

Overall, it can be said that a positive trend of yoga is evident and that patients can benefit in terms of depression and mood through regular yoga. It is not possible to make a clear distinction which aspects of yoga influence the various symptoms of depression and in which way [16]. By monitoring mood separately, no antidepressive effect based solely on mood could be identified, in line with other results [30]. On the contrary, other aspects of depression such as reduction of energy, lack of interest, rumination, or sleep disorders seem to be positively modified by regular yoga. Yoga seems to have a holistic effect and to address various aspects of the mind as well as improving physical parameters associated with mood and depression [16]. Studies have shown that people who regularly practice yoga are more attentive to themselves and their environment, improve sleep patterns and body awareness, have a reduced stress experience and enhance their quality of life [17]. These aspects of self-care and relaxation, non-judgement, self-compassion and overall well-being are known as protective factors from depression research [17, 69, 70]. Through regular yoga practice there is the possibility of transferring the positive aspects and thereby possibly a yoga-induced depression prophylaxis. So far, no adverse effects or side effects have been identified. The positive influence and benefits should be contrasted with possible risks. Yoga provides a low-cost alternative with good accessibility and a fast response in the treatment of MDD.

## Conclusion

The current study follows the trends that have so far appeared in the literature, but it clearly shows that there are various limitations. Depression symptoms can be improved. Also, no adverse effects of yoga appeared in the current study. Especially at the beginning of the yoga practice reduced depression symptoms were measured. Particularly, an increase in positive affect was observed. Nevertheless, a superiority of yoga compared to a TAU could not be identified. Ashtanga-Yoga as a physically demanding and vigorous style needs to be compared with mindfulness-based styles in the future to receive more information about the required physical effort, the frequency and the duration of exercises in the treatment of depression.

## Supplementary Information

Below is the link to the electronic supplementary material.

Supplementary file1 (DOCX 21 kb)

## Footnotes

1 The RS model cannot be calculated as it is not converged. There are no further parameters (*χ*^2^ and *p*) to be reported. The RI model is chosen as the most restrictive model.

2 The RS model cannot be calculated as it is not converged. There are no further parameters (*χ*^2^ and *p*) to be reported. The RI model is chosen as the most restrictive model.
