# Supplementary material for: Effects of body-oriented yoga: a RCT study for patients with major depressive disorder
Source: Eur Arch Psychiatry Clin Neurosci. 2021 Jul 10;271(7):1217–29. doi: 10.1007/s00406-021-01277-5 (PMC8429165; doi:10.1007/s00406-021-01277-5)
Supplement: Supplementary file 1 — Supplementary file1 (DOCX 21 kb) [file 406_2021_1277_MOESM1_ESM.docx]

**Supplementary material:**

1. **final model equation**

*γ* = AV (dependent variable)

Time: measurement points T1, T2, T3

Group: Control vs. Yoga

Random Intercept Model

Level 1: $y= \beta_{0j}+\beta_{1j}*time+r_{ij}$

Level 2: $\beta_{0j}= y_{00}+y_{01}*group+u_{0j}$

$$\beta_{1j}= y_{10}+y_{11}*group$$

Random Slope Modell

Level 1: $y= \beta_{0j}+\beta_{1j}*time+r_{ij}$

Level 2: $\beta_{0j}= y_{00}+y_{01}*group+u_{0j}$

$$\beta_{1j}= y_{10}+y_{11}*group+u_{1j}$$
